# Edible Plant Oil: Global Status, Health Issues, and Perspectives

**DOI:** 10.3389/fpls.2020.01315

**Published:** 2020-08-28

**Authors:** Ying Zhou, Weiwei Zhao, Yong Lai, Baohong Zhang, Dangquan Zhang

**Affiliations:** ^1^Henan Province Engineering Research Center for Forest Biomass Value-added Products, College of Forestry, Henan Agricultural University, Zhengzhou, China; ^2^Department of Biology, East Carolina University, Greenville, NC, United States

**Keywords:** plant oil, environmental factors, oil quality, edible safety, whole-industrial-chain monitoring

## Abstract

Edible plant oil (EPO) is an indispensable nutritional resource for human health. Various cultivars of oil-bearing plants are grown worldwide, and the chemical compositions of different plant oils are diverse. The extremely complex components in oils lead to diverse standards for evaluating the quality and safety of different EPOs. The environment poses great challenges to the EPO safety and quality during the entire industrial chain, including plant cultivation, harvesting, oil processing, and storage. Environmental risk factors include heavy metal or pesticide residue pollution, insect or harmful microbial infestation, and rancidity. Here, the diverse components in oil and various oil-producing processes are discussed, including plant species, oil yield, and composition complexity, environmental factors that degrade oil quality. Additionally, we propose a whole-industrial-chain monitoring system instead of current single-link-monitoring approach by monitoring and tracking the quality and safety of EPOs during the entire process of plant cultivation, raw materials harvest, oil process, and EPOs storage. This will provide guidance for monitoring the quality and safety of EPOs, which were challenged by the deteriorating environment.

**Graphical Abstract:**
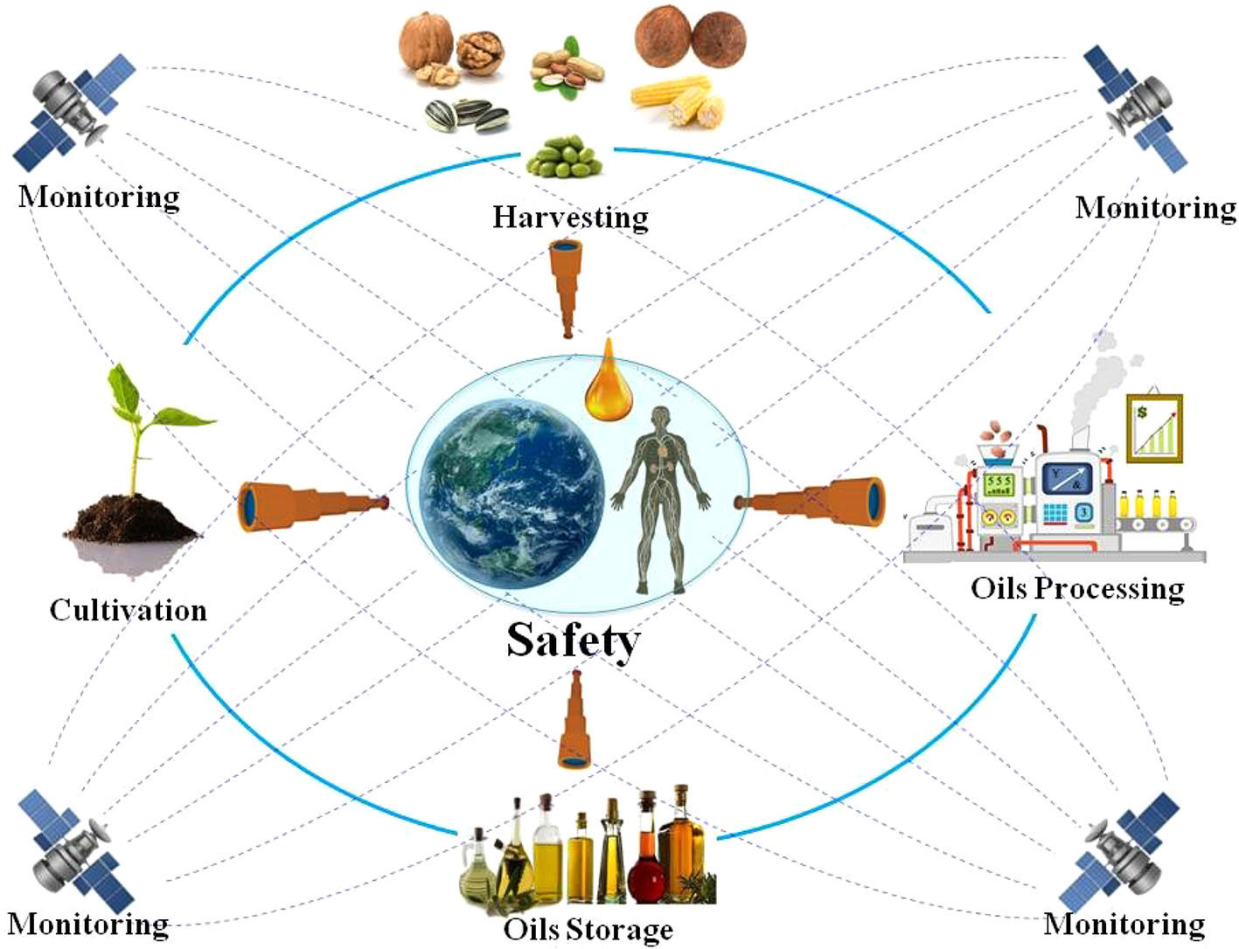
Graphical Abstract The quality and safety of edible plant oil is very important. In the entire production and industrial chain, including cultivation, harvesting, processing, and storage, it is necessary to layers of checks and set evaluation indicators to ensure the quality and safety of edible plant oils, which were challenged by the deteriorating environment.

## Introduction

Edible plant oil (EPO) is obtained from the seeds, pulps, fruits, and plumules of certain plants. As one of the three major energy resources for human life activities, EPO is majorly used in cooking, but also used in a small amount for cosmetics, health supplement capsules, and other purposes. According to the information provided by the U.S. Department of agriculture (USDA), the market of EPO was close to 203 million tons in 2019 (https://www.qianzhan.com/analyst/detail/220/200713-f26ac6c2.html). When used in cooking, oils can change the sensory properties of food such as color, fragrance, and taste in the cooking process, and they also provide diversified flavor and enhance the sense of satiety. As an indispensable part of human dietary nutrition, the safety of cooking oils is paramount to human health. With the increasing of EPO consumption and the aggravation of environmental pollution in recent years, the quality and safety of EPO has gained more attention, and this poses huge challenge to the EPO industry. A strict monitoring system is required throughout the cultivation of oil plants, the processing, transportation, and storage of EPO.

Plant can be used to produce edible oil from their seeds, germs, and/or fruits. In the early human history, sesame (*Sesamum indicum*) oil and olive (*Olea europaea*) oil were commonly used as EPOs. With the development of agriculture, processing and inspection technologies, more and more plants have been developed for EPO production. Many herbaceous plants produce a high percentage of EPO. However, the oil content, composition, and biological activity of different plant species or plant parts greatly vary (**Table 1**). Although sesame has the highest oil content among the herbal oil crops, sesame oil is not a commonly used edible oil because of its low global production and inefficient processing technology (Yi et al., 2017). Nowadays, soybean (*Glycine max*) oil and rapeseed (*Brassica napus*) oil are globally produced in the largest quantities. Additionally, many woody plants are also used for EPO production (**Table 2**). Oil-seed camellia, oil palm, olive, and coconut (*Cocos nucifera*) are the four well-known woody edible oil plants in the world, as they possess a high oil content. Among bulk herbaceous edible oils, the unsaturated fatty acids (UFAs) are the highest, approaching 80%, in peanut oil and rapeseed oil. While among EPOs from woody plants, olive oil and oil-seed camellia (*Camellia oleifera*) oil exceed 80% of UFAs, and camellia oil reaches 90%. Therefore, the EPO quality of most woody plants is better than that of herbaceous plants. With the advantage of no use of cultivated land, the development and utilization of woody oil will play an important role in global grain and edible oil security.

**Table 1:** Herbaceous oil-bearing plants.

No.	Common name	Species	Genus	Familia	Main producing area	Oil content	References
1	Soybean	*Glycine max (Linn.) Merr*.	*Glycine*	*leguminosae* sp.	China, the United States, Brazil et al.	18–24%	(Li et al., 2018)
2	Rape	*Brassica napus* L.	*Brassica*	*Brassicaceae*	All over the world	37.5–46.3%	(Zhao et al., 2005; Ishaq et al., 2017)
3	Sunflower	*Helianthus annuus*	*Helianthus*	*Compositae*	All over the world	46–50%	(Rauf et al., 2017)
4	Peanut	*Arachis hypogaea* L.	*Arachis*	*leguminosae* sp.	Asia, Africa, America, et al.	46–57%	(Wang X. et al., 2018)
5	Cotton	*Gossypium spp*	*Gossypium*	*Malvaceae*	China, the United States, India, Uzbekistan, Egypt, etc.	15–40%	(Shang et al., 2017)
6	Corn	*Zea mays* L.	*Zea*	*Gramineae*	Tropical and temperate regions of the world	4.5–4.8%	(Wang et al., 2010)
7	Sesame	*Sesamum indicum*	*Sesamum*	*Pedaliaceae*	Tropical and temperate regions	43–61%	(Latif and Anwar, 2011)
8	Hemp	*Cannabis sativa L. subsp. sativa*	*Cannabis*	*Moraceae*	All over the world	25–35%	(Vonapartis et al., 2014)
9	Grape	*Vitis vinifera* L.	*Vitis*	*Vitaceae*	All over the world	10–20%	(Movahed and Ghavami, 2007)
10	Fiberflax	*Linum usitatissimum* L.	*Linum*	*Linaceae*	Mediterranean region, Euro-Asian Temperature Zone	35–45%	(Martinchik et al., 2012)
11	Safflower carthamus	*Carthamus tinctorius* L.	*Chelonopsis*	*Labiatae*	China, Russia, Japan, North Korea, et al.	About 40%	(Toma et al., 2014)
12	Rice	*O. sativa*	*Oryza*	*Poaceae*	Almost everywhere, expect Antarctica.	15–23%	(Ju and Vali, 2005)
13	Perilla	*Perilla frutescens (*L*.) Britt*.	*Perilla*	*Labiatae*	India, Myanmar, Japan, Korea, Indonesia, Russia, et al.	40–50%	(Liao et al., 2018)

**Table 2:** Woody oil-bearing plants.

No.	Common name	Species	Genus	Familia	Main producing area	Oil content	References
1	Oil palm	*Elaeis guineensis* Jacq.	*Elaeis*	*Arecaceae*	Tropical regions of Africa, tropical regions of China, Taiwan, Hainan and Yunnan.	50–55%	(Kasemsumran et al., 2012)
2	Coconut	*Cocos nucifera* L.	*Cocos*	*Arecaceae*	Asia, Africa and Latin America	65–74%	(Marina et al., 2009)
3	Olive	*Olea europaea* L.	*Olea*	*Oleaceae*	Mediterranean coast	31–56%	(Sun et al., 2017; Olmo-García et al., 2018)
4	Tea-oil tree	*Camellia oleifera Abel*	*Camellia*	*Theaceae*	From Yangtze River Valley to Southern China	47.0–59.5%	(Chen et al., 2011)
5	Walnut	*Juglans regia* L.	*Juglans L*.	*Juglandaceae*	Southeastern Europe, Himalaya mountains, China	50–70%	(Özcan et al., 2010)
6	Peony	*Paeonia suffruticosa* Andr	*Paeonia*	*Paeoniaceae*	Henan, Sichuan, Tibet, Guizhou, Yunnan of China	27–33%	(Ning et al., 2015; Zhang et al., 2018)
7	Pecan	*Carya cathayensis* Sarg.	*Carya*	*Juglandaceae*	Anhui and Zhejiang, China	60–70%	(Huang et al., 2016)
8	Hazelnut	*Corylus heterophylla* Fisch.	*Corylus*	*Betulaceae*	Temperate zone in Asia, Europe and North America	50–75%	(Balta et al., 2006; Miraliakbari and Shahidi, 2008; Juhaimi et al., 2018)
9	Idesia	*Idesia polycarpa* Maxim.	*Idesia*	*Flacourtiaceae*	Southwest China, North Korea, South Japan.	21.2–44.0%	(Zhu, 2010; Gong et al., 2012; Li R. J. et al., 2016)
10	Pine	*Pinus*	*Pinus*	*Pinaceae*	Brazil, coniferous forests, et al.	58–69%	(Ryan et al., 2006; Bao and Guo, 2016)
11	Cocoa	*Theobroma cacao* L.	*Theobroma*	*Sterculiaceae*	Narrower within 10°north-south latitude of the equator	45–60%	(Servent et al., 2018)
12	Shiny-leaved yellowhorn	*Xanthoceras sorbifolium Bunge*	*Xanthoceras Bunge*	*Sapindaceae*	North and northeast China	50–60%	(Cao, 2015)
13	Acer truncatum	*Acer truncatum* Bunge	*Acer Linn*.	*Aceraceae*	Northeast and north China, Shaanxi, Sichuan, et al.	42–46%	(Zhang and Hou, 2010)

Here, we review the distribution of edible oil-bearing plants in the world and the complicated chemical composition of EPO, including their importance for our health. This review will also focus on the environmental risks during the process of EPO, from the cultivation of oil-bearing plants and the harvest of raw materials to the production and storage of EPO. The industrial chain monitoring system for the quality and safety of EPO is also proposed.

### Global Production of EPO

The yield of oil-bearing plants is the guarantee of the source of EPO. As the growing of global population, the demand for EPO is also on the rise and many oil-bearing plants are being grown on a large scale, widely using agricultural machinery and high-tech methods, such as cell engineering, genome editing, and tissue culture. As more attraction to human health, people are preferred to consume more healthy oils with higher UFAs, such as olive oil, walnut oil, and corn oil (Weinstock et al., 2006; Geng et al., 2018; Wang et al., 2011; Lin et al., 2018). In China, camellia oil and olive oil have maintained a high growth rate, significantly higher than the overall growth rate of the domestic oil industry.

**Figure 1:**
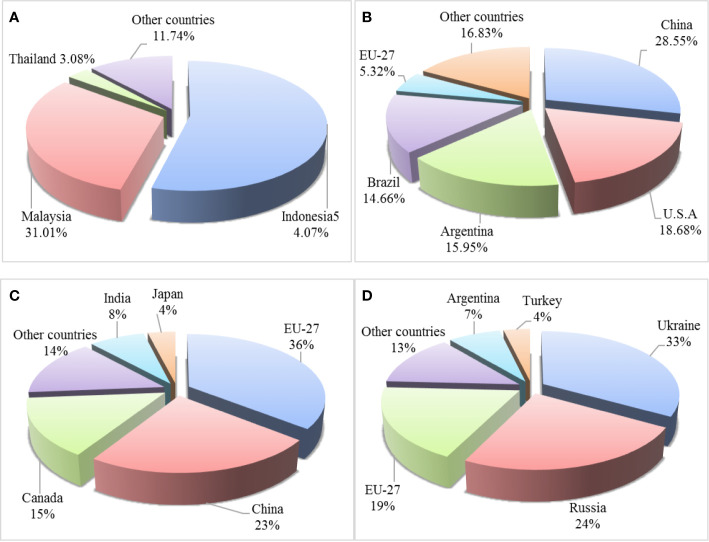
Major countries producing palm oil **(A)**, soybean oil **(B)**, rapeseed oil **(C)**, and sunflower seed oil **(D)** in 2016.

Among the common EPOs, palm oil has the highest annual yield, showing an increased trend in recent years. Soybean oil is with the second highest annual yield, followed by rapeseed oil (https://www.qianzhan.com/analyst/detail/220/200713-f26ac6c2.html). Overall, the global production of major EPO is increasing annually (**Table 3**). Palm oil is mainly produced in southeastern Asia, including Malaysia and Indonesia (**Figure 1A**) (Esteki et al., 2018). In 2016, palm oil from Indonesia and Malaysia was accounted for approximately 85% of global palm oil production, including palm kernel oil. The main producers of soybean oil are China, the United States, Argentina, and Brazil, among which China’s soybean oil production ranks first in the world (**Figure 1B**). The European Union, China, and Canada are the largest producers of rapeseed oil. In 2016, the European Union led the world in rapeseed oil production, accounting for 35% of the total, followed by China in which accounted for approximately 23% of the total yield (**Figure 1C**). Olive oil is mainly produced by the Mediterranean coastal countries, including Spain, Italy, Greece, and Turkey. These countries are account for 90% of the world’s total olive oil production, of which Spanish olive oil production ranks the first in the world. In southeast Asian countries, Philippines is rich in coconut oil. China and India are major producers of peanut oil, and Ukraine is the world’s largest producer of sunflower seed oil (**Figure 1D**).

**Table 3:** Global yield of common EPOs during 2012 to 2018 from U.S. Department of Agriculture (USDA) (millions tons).

	2012	2013	2014	2015	2016	2017	2018
Coconut oil	3.43	3.65	3.37	3.32	3.39	3.54	4.09
Cottonseed oil	5.24	5.22	5.12	4.29	4.42	5.16	4.09
Olive oil	3.46	2.44	2.40	3.13	2.48	3.27	2.04
Palm oil	52.58	56.42	61.75	58.89	65.26	69.28	73.58
Palm kernel oil	6.16	6.63	7.32	7.00	7.63	8.17	8.18
Peanut oil	5.29	5.51	5.44	5.48	5.96	6.09	6.13
Rapeseed oil	24.01	24.83	27.53	27.77	28.17	28.48	28.61
Soybean oil	42.73	43.07	49.28	51.53	53.68	54.95	57.23
Sunflower seed oil	14.34	12.87	14.97	15.38	18.18	18.37	20.44
Total	157.24	160.64	177.17	176.78	189.17	197.32	204.39

## Complexity of the Chemical Composition of EPOs

EPOs contain complex chemical components, and are generally rich in fatty acids, microelements and active compounds, and flavor substances (Kim et al., 2010; Puch et al., 2010; Ascensión et al., 2014; Wang et al., 2019). These components together constitute the unique physicochemical properties of EPOs. EPOs are also rich in the fat-soluble vitamins A, D, E, and K, among which vitamin E has antioxidant properties and can devour the free radicals that lead to aging and carcinogenesis.

### Fatty Acid Composition in EPOs

Fatty acids are the major composition of oils. A fatty acid is an organic substance consisting of a long aliphatic hydrocarbon chain that contains one carboxyl group at one end. Fatty acids are divided into saturated fatty acids (SFAs) and UFAs (Esteki et al., 2018). The human body can synthesize the required SFAs and UFAs with only one double bond. Fatty acids containing two or more double bonds must be obtained from the diet, and therefore, the latter are called essential fatty acids, among which linolenic acid and linoleic acid are the most important.

UFAs play important roles in the human body, such as maintaining the relative fluidity of cell membranes to ensure the normal physiological function of cells, esterifying cholesterol, and reducing cholesterol and triglyceride in the blood (Assmann et al., 2018). They are the precursors of prostaglandin synthesis, reducing blood viscosity, increasing blood microcirculation and the activity of brain cells, and enhancing memory and thought processes. There are 16 or 18 carbon atoms in the most abundant fatty acids, which are oleic acid, linoleic acid, linolenic acid, and SFA. However, different oils have different fatty acid compositions (**Table 4**).

**Table 4:** Fatty acid content (%) in some EPOs.

Edible plant oils	Saturated fatty acids (SFAs)	Unsaturated fatty acids (UFAs)			Reference
		Oleic acid	Linoleic acids	Linolenic acids	
Soybean	6.0–24.0%	15–36%	42.8–56.1%	2–14%	(Wijewardana et al., 2019)
Rapeseed	6.46–9.43%	56.0–72.0%	13.8–24.6%	4.3–11.3%	(Pospišil et al., 2007)
Sunflower seed	9–13%	16.4–27.6%	60.2–72.1%	0.07–1.8%	(Orsavova et al., 2015; Wei and Wang, 2017)
Peanut	9.9–13.8%	37.0–55.6%	25.3–39.7%	0.40–3.2%	(Sui et al., 2018)
Cottonseed	27.5–33.7%	16.5–27.0%	43.2–54.0%	0.13–0.3%	(Talpur et al., 2014; Wang et al., 2016)
Corn	15–16%	27.6–34.6%	48.6–55.3%	0.60–1.49%	(Weinstock et al., 2006)
Sesame	12.4–14.4%	36.7–42.94%	43.2–48.6	0.2–0.95%	(Hashempour-Baltork et al., 2018; Ghosh et al., 2019)
Palm	49.7–57.5%	37.3–40.8%	9.1–11.0%	0.01–0.25%	(Nehdi and Sbihi, 2018)
Coconut	81.2–94%	5–10%	1–2.5%	0.2–2.5%	(Xiao and Xia, 2019)
Olive	12.5–20.9%	54.5–80.2%	4.9–21.2%	0.7–1.5%	(Geng et al., 2018)
Camellia	7.7–12.9%	74.3–83.6%	7.0–15%	0.2–0.4%	(Wang et al., 2011; Lin et al., 2018)
Walnut	5.0–17%	10–20%	55–70%	10–18%	(Hayes et al., 2016; Sena-Moreno et al., 2016)
Peony seed	6.2–12.4%	20.5–45.1%	16.5–33.6%	28.1–46.9%	(Llorent-Martinez et al., 2011; Wei and Shao, 2018)

### Trace Components in EPOs

**Figure 2:**
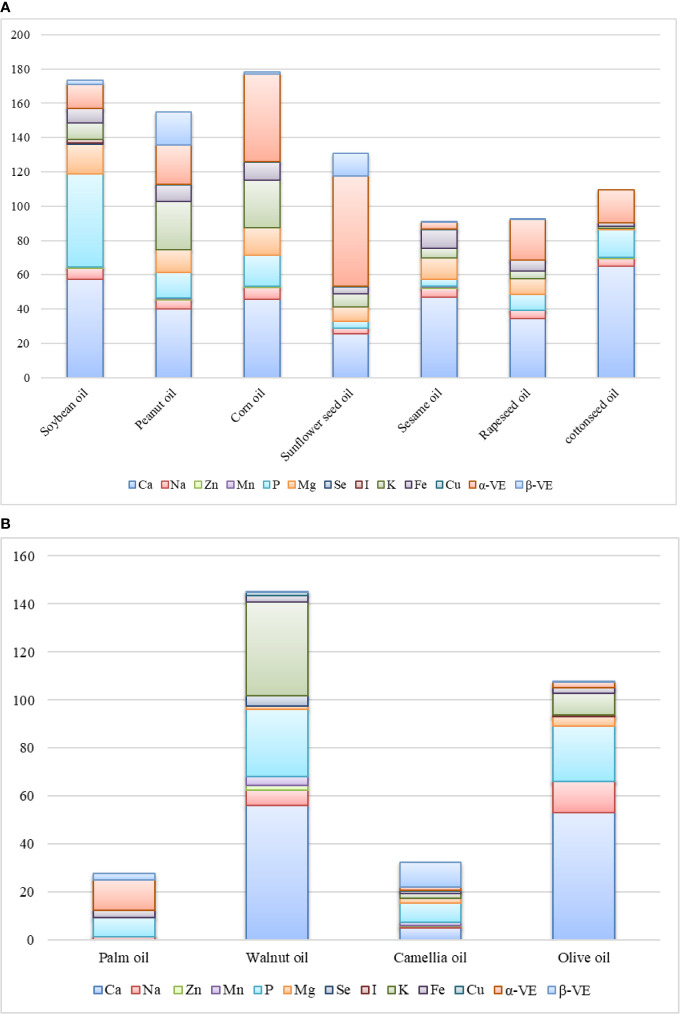
Microactive compositions in **(A)** herbal edible plant oils (mg/100 g) and **(B)** woody edible plant oils (mg/100g).

Many kinds of trace components have been detected in EPOs and their contents vary in different EPOs (**Figure 2**). With the development of detection technology, more and more trace components in EPOs will be characterized.

There are small quantities of trace elements in the human body that are necessary for human survival and health. Because the human body cannot automatically synthesize trace elements, they must be obtained from the diet, for example the EPOs (Llorent-Martinez et al., 2011).

The content of trace elements and active compounds in EPOs is very low, but their biological activities have some unique health functions that are very meaningful.

High quantities of phosphorus (P), magnesium (Mg), and potassium (K) are found in soybean oil (**Figure 2A**). Walnut oil also contains high amounts of P (Juranović Cindrić et al., 2018). P protects human tissue cells and enhances the role of cell membranes. P boosts the effectiveness of the vitamin B family. Phospholipids are formed when P combines with fat in the blood, and this compound plays a structural and metabolic role in the human cell membrane and plays a role in the body tissue structure (Taketani et al., 2015).

Olive oil contains the largest quantities of calcium (Ca) at 53 mg/100 g (Gouvinhas et al., 2015). The function of Ca is to maintain strong bones and healthy teeth, to maintain regular heart rhythms, to relieve symptoms of insomnia, to assist with the metabolism of Fe in the body, and to strengthen the nervous system, especially its stimulating communication function (Marsh et al., 2015).

EPOs also contain other trace elements, including Cu, Zn, and Mn, which play important roles in the development and function of the hair, skin, bone tissue, brain, liver, heart, and other internal organs (Haase and Rink, 2014; Marsh et al., 2015; Schofield, 2017). Most common EPOs contain Cu, except palm oil, but the content of Cu in different EPOs is significantly different. Soybean oil, walnut oil, and olive oil contain selenium (Se) and/or iodine (I), which are not found in other EPOs (**Figure 2**).

The content of alpha-tocopherol (α-VE) in sunflower seed oil and corn oil is 64.12 mg/100 g and 50.94 mg/100 g, respectively (**Figure 2**). α-VE is a very important vasodilator and anticoagulant, which can reduce wrinkles and the oxygen consumption of cells and further help to reduce leg cramps and hand and foot stiffness (Jiang, 2017).

The majority of oils also contain a small amount (0.1–1.0%) of saturated and unsaturated hydrocarbons. For example, squalene, molecular formula C_30_H_50_, is a colorless oily liquid with a pleasant odor; after oxygen absorption, it becomes viscous linseed oil. Squalene was found in some herbaceous and woody oils. Squalene has strong biological activity that robustly transports reactive oxygen species in the blood, enhances the body physiological functions, improves human immunity, and helps the cells resist ultraviolet rays. Squalene is widely used in various fields such as medicine, beauty, and cosmetics. International research has been carried out on the exploration of natural resources and chemical synthesis methods so that squalene can be produced without killing sharks (Kim and Karadeniz, 2012). It is found that the content of squalene in woody oil is higher than that in herbaceous oil.

Among all plant oils, corn oil contains the highest quantities of phytosterols, followed by rapeseed oil (Zou et al., 2018). Phytosterols consist of a variety of ingredients such as β-glutosterol, oil sterol, rapeseed sterol, and soya sterol. Phytosterols can inhibit the absorption of cholesterol, have significant preventive and therapeutic effects on cardiovascular diseases and cervical cancer, and also have strong anti-inflammatory effects (Wang F. C. et al., 2017). Therefore, eating EPO with higher content of phytosterols is a healthy choice.

Carotenoids have been found in soybean and rapeseeds (Syed and Shinwari, 2016). Both chlorophyll A and B are photosensitive substances and are the source of photooxidation of oils (Tena et al., 2018). However, under the condition of no light, chlorophyll A and chlorophyll B capture the free radicals produced during the initial stage of oil oxidation, which subsequently inhibits the automatic oxidation of oils. Particularly, chlorophyll A has a strong antioxidant effect, but different oxidation conditions such as substrate and temperature can affect chlorophyll antioxidant action, even with stronger antioxidant effects at low temperatures (Bhattacharya et al., 2017). This may be one of the reasons for the long storage time of soybean oil and rapeseed oil.

Polyphenols, also known as tannins, are found in herceous edible oils and are one of antioxidant components (Wang X. et al., 2017). It is a generic term for plant components containing multiple hydroxyphenols in their molecules. Polyphenols have strong antioxidant activity in oils and fats and display anti-cancer activity. Polyphenols are also used to ameliorate radiation damage (Rueda et al., 2016).

Flavonoids are a group of substances that contain alpha-phenyl phenylthiopyranone or beta-phenylthiopyranone. Flavonoids are beneficial to the body and act as antioxidants (Cassidy and Minihane, 2017). At present, flavonoids have been found in many EPOs, such as soybean oil, rapeseed oil, and corn oil (Li X. J. et al., 2016). The trace substance of herbaceous and woody oils is basically the same, but the content is different.

### Flavor Substances in EPOs

EPOs are important raw materials in our daily consumption of food, as well as in food processing. They not only provide heat energy and essential fatty acids for human beings but also endow food with a pleasant flavor (Tekaya et al., 2018). With the improvement in living standards, in addition to containing the necessary nutrients in EPOs, the flavor is a major factor that is taken into account when choosing an EPO.

Flavor substances are one of the important indicators of the sensory quality of EPOs (Fujikawa et al., 2002). The unique flavor of different EPOs is not formed by one or several compounds but is formed by the synergy of various components (Zhang et al., 2012; Yang et al., 2015; Liu et al., 2017). Phenols, including tocopherols, polyphenols, phytosterols, and pigments, are important components of natural vegetable oils. Although the contents of these substances are relatively low, they are closely related to the quality of edible oil, which directly affects the functionality and oxidation stability of edible oil. It was found through comparison that the flavor substances in EPOs mainly include alcohols, aldehydes, ketones, alkanes, alkenes, and furans, and the variety and content of flavor components in one EPO is different from each other (Zhang et al., 2012). The volatile flavors of tea oil, olive oil, soybean oil, corn oil, peanut oil, sunflower oil, sesame oil, and rapeseed oil were compared using solid phase micro-extraction-mass spectrometry, and it was found that olive oil contained the largest amount of esters, and the other EPOs had high amounts of aldehyde (Hu et al., 2018).

**Figure 3:**
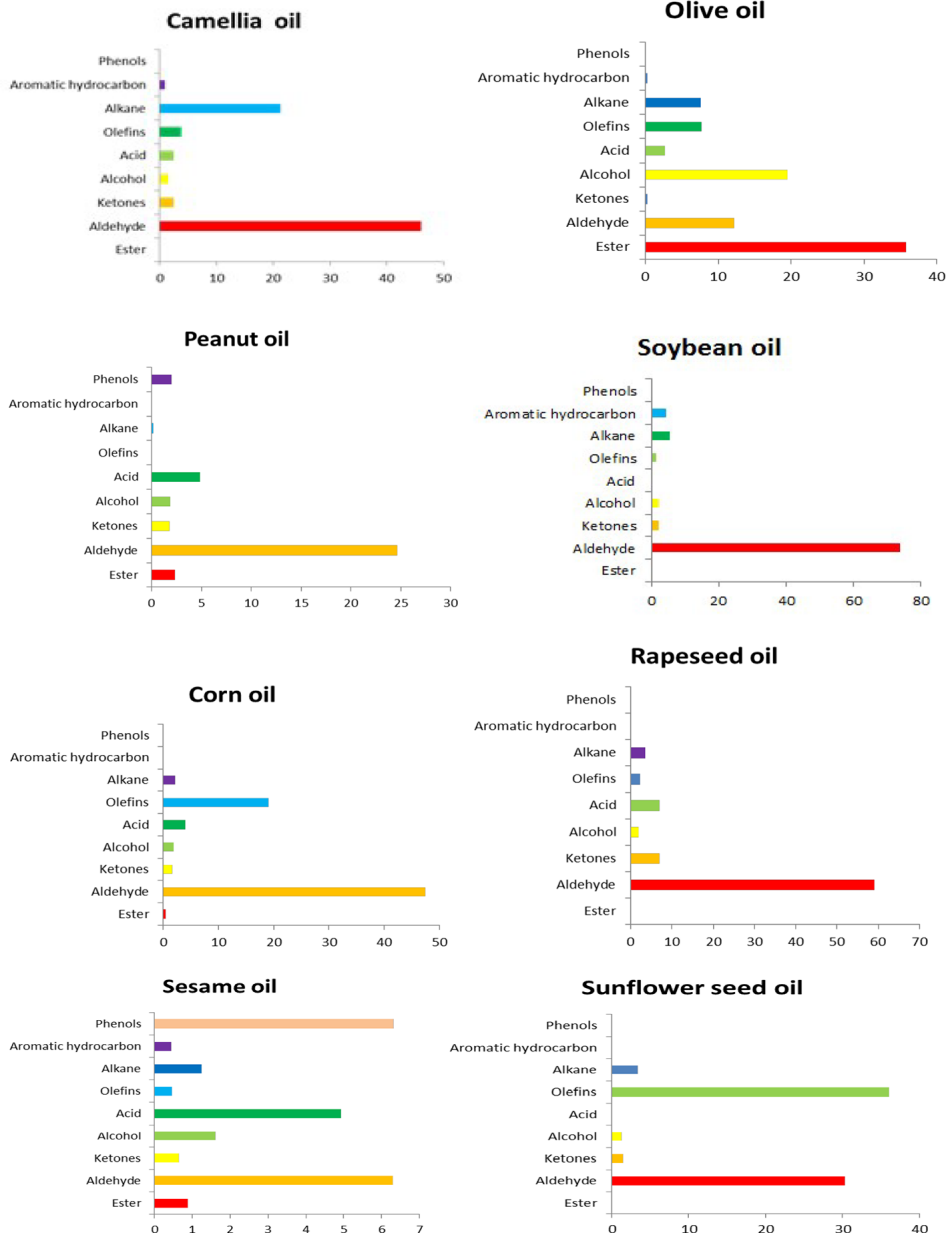
A comparison of different flavor substances in EPOs (%).

As an important flavor substance in oils and fats, esters are easily decomposed or oxidized by heat to form aldehydes and other volatile short-chain secondary oxidation products. No olefin substances were detected in peanut oil. Acidic substances were not detected in sunflower oil or soybean oil. Phenolic substances were only found in peanut oil and sesame oil. All these results indicated that the type and content of flavor substances in different EPOs are not the same (**Figure 3**).

### Hazardous Substances in EPOs

Due to improper cultivation, process, and storage, some hazardous substances are also detected in EPOs. These substances can be divided into two types: (i) biological hazards and (ii) harmful chemical substances (Ji et al., 2016).

For EPOs, biological hazards usually arise from the oil-bearing plants that are infected by microorganisms or infested by pests. During plant growing, raw materials harvesting and storing, microorganisms, parasites, and insects attack plants, destroy their epidermis, and leave harmful secretions in or on the surface of seeds or fruits, contaminating the raw materials of EPO process (Bhat and Reddy, 2016; Zhou et al., 2017).

The majority of harmful chemical components detected in EPOs come from pesticide residues, heavy metals, or plasticizers (Hu et al., 2016). It should also be noted that in the process of cooking with edible oils, high temperature will change the structure of the oil and produce harmful trans-fatty acids, which are harmful to human health (Ginter and Simko, 2016). Improper use of storage containers can also cause some harmful substances to enter the EPOs, such as some plasticizers and submicrometre plastics from plastic containers, which may pose a health threat if they enter the internal circulation of human body. Heavy metals such as lead in glass containers can also contaminate EPOs (Li et al., 2013; Sungur et al., 2015).

## EPOs Affected by Environmental Risks

**Figure 4:**
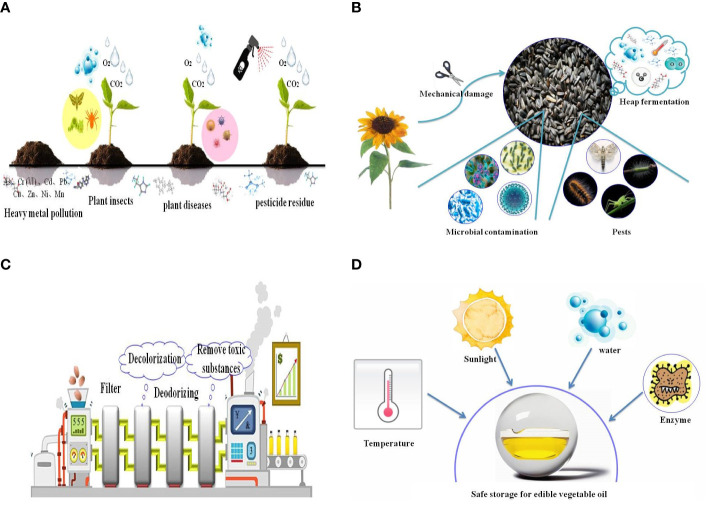
Environmental risks in **(A)** oil-bearing plant cultivation, **(B)** the harvesting and post-harvest process of EPOs, **(C)** during EPO processing, and **(D)** during EPO storage. The graph takes sunflower seeds as an example to show the risks that oil crops will encounter during harvesting and post-harvesting process.

Oil-bearing plants are affected by various external environmental factors, such as heavy metals, pests, and diseases, and pesticides and herbicides used during plant growth and development, which will directly affect the safety of EPOs. With the continuous development of society, there has been increasing concern over the quality and safety of edible oils. The requirements for the quality of oil-bearing plants are also increasing. The improvement of the quality of oil-bearing plants has become a necessary factor for modern edible oil production. Therefore, in order to fundamentally improve the quality of oil-bearing plants, it is necessary to comprehensively analyze the planting safety of oil-bearing plants (**Figure 4A**).

### Risks During Oil-Bearing Plant Cultivation

At present, oil-bearing plants are threatened by diseases, pests, air and soil pollution, pesticide residues, and other environmental factors (Wang Y. et al., 2018). The major pollution affecting oil quality and safety is heavy metals and pesticides. Many contaminants have been identified during the planting stage that will enter into the vegetable oil. Some fungi can infect oil plants and produced large amounts of toxins that are very harmful to our human body. *Aspergillus flavus* and *A. parasiticus* can infect peanuts causing a severe decline in yield, and what’s more, aflatoxin produced by them is serious carcinogen (Zhang et al., 2017).

Pesticides play an important role in controlling diseases and pests of oil plants, but at the same time, they also pollute the environment and oil plants. During oil processing, pesticides will migrate and eventually accumulate in edible oils. Pesticide residues in EPOs have become a major factor that adversely affects human health (Wei et al., 2008; Yao, 2016). Common pesticides are endosulfan, chlorpyrifos, cypermethrin, and HCH, classified as persistent organic pollutants because of their persistence, bioaccumulation, long-distance migration, and adverse effects on organisms (Frigo et al., 2002). Some studies suggest that the absorption and accumulation of organic pollutants by plants depends not only on the nature of the pollutants but also on the oil content of oil plants. Oil plants with higher oil content absorbed more lipophilic substances such as toxaphene (McLachlan, 1996; Wei et al., 2006).

Most oil-bearing plants become contaminated with heavy metals such as As, Cr (VI), Cd, Pb, Cu, Zn, Ni, Hg, and Mn during their growth stage, which will severely affect the safety of edible oil (Xue et al., 2019). When heavy metals sufficiently accumulate in cultivated soil, they can directly injure crop growth, resulting in reduced yield and quality (Sun et al., 2016). More seriously, heavy metal can be absorbed into the plant cells and contaminate the oils. In order to ensure the supply safety of oil-bearing raw materials, it is necessary to perform sufficient testing and treatment technology in order to reduce the safety hazards caused by edible oil raw materials in the planting stage.

In addition, with the extensive use of plastic products, micro-plastics have been detected in various ecological environments around the world, which has aroused widespread concern. It has been proved that microplastics could been absorbed by plants and affected their growth, which posed a potential risk to our food safety (Li et al., 2020; Sun et al., 2020). Microplastics may enter the human body through oil-bearing plants and pose a threat to human health, and this risk will be exacerbated due to the adsorption properties of microplastics to heavy metal ions (Mao et al., 2020; Yu et al., 2020).

### Risks During Harvesting and Post-Harvest

After raw materials are harvested, they may not be immediately pressed into EPOs, so they often need to be stored for a period of time (**Figure 4B**). Some oil-bearing plants have a higher oil content, especially sunflower seeds, with an oil content of 45 to 60% and UFA content of more than 90%, which is prone to heat, mildew, oil rancidity, and deterioration during storage (Mangin et al., 2017). It is difficult to store sunflower seeds without spoilage, and advanced technology is required for preservation. At present, the methods for preserving and storing vegetable oil raw materials include physical preservation techniques such as cryopreservation, cold shock-ultraviolet and microwave irradiation, and chemical preservatives such as thiophanate, diazolid, and carbendazim. However, these chemical preservatives will also pollute oil raw materials.

Water and temperature play a dominant role in seed storage (Cheong et al., 2018). The moisture content of the seeds affects the preservation time and quality of EPO raw materials. Seeds should be fully dried before storage because water causes deterioration. Storage sites should be kept ventilated and dry, strictly disinfected, sterilized, and moisture-proof., and it is important to remove any impurities, rotten fruit, or mildew-contaminated seeds. The preservation methods for various EPO raw materials will be different, but the ultimate goal is to ensure that the raw materials do not deteriorate and prevent various mildew reactions. If the raw materials are rancid and deteriorated and contain molds, then they cannot be used in edible oil production. It has been reported that with the prolongation of storage time, the quality and yield of oil from the seeds was decreased (Bonte et al., 2017; Han et al., 2017).

### Risks During Oil Processing

Pressed extraction of EPOs is the most traditional way to extract oil, and it is also the most widely used method currently (Yara-Varon et al., 2017). Compared with solvent extraction, pressed extraction has the advantages of simple operation, no solvent pollution, high quality of crushed oil, and retention of the unique flavor of EPOs (**Figure 4C**). Pressing extraction of edible oil can be divided into hot-pressed and cold-pressed (Pereira et al., 2014). Cold-pressed is a method of producing oil by low-temperature pressing through an oil mill. The oil plants are not heated, nor are they are stir-fried at low temperature before pressing. During extraction at the oil mill, the entire pressing process is performed at low temperatures, which ensures that the oil will contain the maximum amount of flavor substances (Fawzy, 2013; Yang et al., 2013; Sielicka et al., 2014). The hot-pressed method is the opposite, and the oil pressing process occurs at a continuous high temperature environment (Siger et al., 2017). At high temperatures, oil plants can increase oxidation stability, but hot-pressing also has some drawbacks. High temperature may lead to loss of many nutrients and flavor substances in EPOs (Soria and Villamiel, 2010; Teh and Birch, 2014; Koubaa et al., 2016). For example, high temperature caused vitamin E damage and loss. Research showed that the content of vitamin E in cold-pressed and hot-pressed sunflower seed oil was 45.7 mg/100 g and 13.2 mg/100 g, respectively. This indicates that vitamin E decomposed to a certain extent during the baking process, thus resulting in a decrease in the vitamin E content (Wei and Wang, 2017). Therefore, cold-pressed method becomes more popular.

### Risks During Oil Storage

Improper storage conditions or long term of storage decreased the quality of EPOs (**Figure 4D**). Severe deterioration of EPOs will result in adverse effects on human health, and lose their edible value. Conditions that deteriorate EPOs are light, heat, oxygen in the air, water, and enzymes in oils, and the high UFA content of some EPOs requires additional storage measures to ensure safety (Jabeur et al., 2015). Consequently, storage conditions are very important in the storage process. If the standard of containers for EPOs is not strict, EPOs will be contaminated with plasticizers, heavy metal, synthetic antioxidants (Carvalho et al., 2019), or other toxic substances during storage.

The types of rancidity are oxidative rancidity, hydrolytic rancidity, and ketone rancidity (Haman et al., 2017). Although the processes of the rancidities are different, any rancidity will eventually lead to the production of a certain amount of alcohols, aldehydes, and ketones, which will cause serious harm to the consumer. Severe rancidity will be accompanied by a pungent unpleasant smell due to the aldehydes, ketones, and other oxides. The fatty acid composition of the oil directly affects the shelf life of EPOs (Buratti et al., 2018). SFAs must be acidified by enzymes or fungi, or conditions must be optimal for the existence of hydrogen peroxide, and therefore, oils with a higher content of UFAs are more likely to be acidified, which poses a challenge to store EPOs with high-quality.

The physical factors that affect the storage safety of EPOs are heat, water, oxygen, and other factors. The rate of the rancidity reaction increases one fold with an increase in storage temperature of 10°C, and therefore, the better choice is to maintain the process of oils production and storage at a low temperature. The effect of the moisture content in oils is very complex on the oxidation of oils, and the moisture content also affects the growth of microorganisms that can cause the spoilage of oils. Rancidity can be prevented by reducing the water content through refining and dehydration. Oxygen plays an important role in rancidity because the higher the oxygen content, the faster the occurrence of ketone rancidity and oxidation rancidity. Oxygen can be removed by adding nitrogen to filled bottles and vacuum packaging or adding antioxidants to reduce the oxygen content of edible oils. During the storage of edible oil, direct sunlight and metal contact should be avoided, because radiation can significantly increase the rate of free radical formation and increase the sensitivity of fatty acid oxidation; metal ions can catalyze the oxidation of oil and greatly increase the decomposition rate of hydrogen peroxide.

The safety of edible oil storage is also affected by chemical factors. Some edible oils are naturally pigmented, and the pigments easily form pigmented peroxide complexes, thus accelerating the degradation of oils (Wang H. C. et al., 2018). The addition of antioxidants to oils will increase the shelf life of edible oils. At present, the main synthetic antioxidants are BHA, BHT, PG, TBHQ, and THBP (Cini et al., 2014; Kim et al., 2016). Natural antioxidants mainly include vitamin C, vitamin E, carotenoids, and polyphenols such as flavonoids and tea polyphenols. In addition, rosemary extract as a natural antioxidant has been accepted by more and more people. Rosemary extract significantly improved the scavenging ability of free radicals in oil, delayed deterioration, and extended the shelf life of oils (Yang et al., 2016; Bañares et al., 2019).

## Industrial Chain Monitoring the Quality and Safety of EPOs

Edible oil industry is a complicate supplier chain, which is involved in plant planting, seed storage, transportation, production, processing, oil storage, and transportation. These links are interrelated, mutually restrictive, and interlinked. Security problems taking place in any link will affect the EPO quality. Therefore, in order to ensure the safety of edible oil, we must seize every link of the edible oil industry and take proper safety measures to monitor the entire process. A strict and reasonable evaluation system is the key to ensure the quality and safety of EPOs.

### Monitoring During Cultivation Stage

During cultivation stage, oil-bearing plants mainly encounter three major elements: natural environment, pests and diseases, and exposure to chemical pesticides. Natural environment includes air, water, light and soil, while atmospheric pollution mainly includes inhalable particulate matter, SO_2_, and nitrogen oxides. Some studies have shown that SO_2_ can affect carbohydrate synthesis in plants, which causes plant cell membrane lipid peroxidation, permeability damage, ion exosmosis, and the increase of ethylene production *in vivo* before injury, leading to premature maturation and senescence of plants. This is a great damage to oil crops at seedling stage and eventually affects the yield of oil plants. Thus, it is important to monitor the air quality in the growing environment of oil plants.

Soil is the basis of providing nutrients for plant growth and development, and therefore, good soil conditions are required for growing oil-bearing plants. It is necessary to strengthen soil quality monitoring. Oil-bearing plants inevitably encounter diseases, insect pests, and weeds during their growth and development, which not only affect plant growth but also affect the quality of fruit and ultimately affect the quality of EPOs. In India, the fungus *Sclerotinia sclerotiorum* occurred on soybean and caused an approximately 40% reduction in soybean yield (Wrather et al., 2001). Pesticides are effective measures to control the effects of pests and weeds. The use of pesticides can increase yield and economic benefits, but it also affects the soil and fruit. Therefore, we should strictly control the soil conditions, the degree of pests and diseases, and the use of pesticides. Common control technologies of pest and disease are as follows (Li and Zhang, 2018):

1. Biological control and the use of pesticides/fungicides are effective preventive measures for eliminating hidden dangers over time.
2. Various cultivation modes such as rotation and interplanting can be adopted.
3. Using resistant varieties under good cultivation management can enhance plant resistance to stress.
4. New technologies and methods can be used to identify pests and diseases more rapidly.

To solve the key problems of pesticide residues, it is important to establish a good evaluation system (Li, 2002). It has been reported that the greater the amount of pesticides sprayed, the better the control of pests/diseases and the higher the yield (Gagic et al., 2017), but the more the amount of pesticide residues, the accumulation of pesticide residues to a certain amount will cause great harm to human and the environment. It is best to use environmentally friendly pesticides with excellent control, little impact on natural enemies, low toxicity, and no residues. The use of chemical pesticides should be strictly controlled (Cao et al., 2018). There are many types of pesticides, and the standards of pesticide usage vary in different countries. Therefore, it is necessary to establish a strict and uniform evaluation criterion for the usage of pesticides and pesticide residues.

Heavy metals not only pollute the soil but also negatively affect food safety when plants are contaminated with the heavy metals from the soil (Fryzova et al., 2018). Therefore, it is necessary to control the amounts of heavy metals in the soil. It is indispensable to establish relevant soil heavy metal standards and limits (**Table 5**) and regularly monitor the content of heavy metals in the soil where oil plants are planted. Once the content of heavy metals in soil exceeds the standard, the cultivation of oil plants should be prohibited. If the soil is slightly polluted by heavy metals, the oil plants with weak adsorption capacity should be selected. Furthermore, it is still necessary to monitor the content of heavy metals in processing raw materials. The varieties with strong resistance should be selected before planting to reduce the amount of pesticide. Some genetically modified oil crops may have strong resistance and weak adsorption capacity of heavy metals. However, the safety of genetically modified organisms is still controversial. Thus, the supervision of genetically modified oil crops must be in place, and the products may be clearly marked.

**Table 5:** Grade evaluation of heavy metal pollution in soils (mg/kg).

	Hg	Pb	Cd	Cr	As	Cu	Zn	Reference
Clear area	0.3≤	25≤	0.2≤	34≤	10≤	32≤	74≤	geochemistry background value (level I)
Passable region	0.4≤	80≤	0.3≤	50≤	15≤	124≤	106≤	Canadian Soil Quality Guidelines (level II)
Slight pollution	0.5≤	100≤	0.4≤	100≤	20≤	216≤	237≤	Soil Screening Levels (SSLs)
Moderate pollution	0.6≤	140≤	0.6≤	200≤	25≤	308≤	343≤	Soil Guideline Values (SGV)
Heavy pollution	1.0≤	240≤	0.8≤	300≤	30≤	400≤	500≤	Soil Environmental Quality Risk control standard for contamination of agricultural land (GB15618-2018)

### Monitoring During Plant Harvesting Stage

Fruits and seeds are the most common parts of oil plants used for oil extraction. Due to the damage by animal encroachment or machine, a proper harvesting method should be selected during harvesting and a process of checking the integrity of the seeds or fruits is necessary after harvesting. Because damaged seeds and fruits are easier infected by microorganisms, reducing the quality of raw materials. For each kind of oil plants, the exact harvesting time should be firstly considered. Early harvesting may result in a lower oil content in seeds, while late harvesting may reduce yields as some mature seeds fall off from the plants (Matthäus et al., 2018). After harvesting, when the seeds and fruits are not immediately pressed, they need to be carefully stored in the most optimal environment. The main factors affecting the safe storage of oil are moisture, temperature, relative humidity, pests, microorganisms (Mmongoyo et al., 2017; Sobolev et al., 2019), and so on. These factors must be detected frequently during seeds and fruits storage. The evaluation of the harvest mainly includes a survey of the degree of damage to seeds and fruits during harvesting, and storage conditions should be strictly controlled.

### Monitoring During Oil Processing Stage

EPOs can be obtained from the processing of plant seeds or fruits. At present, there are three main processing methods: hot pressing, cold pressing, and solvent extraction; each of the method has its own advantages and disadvantages. Compared with other two methods, cold pressing method maybe the better oil pressing method for obtaining high quality oil. In addition, there is also a method called supercritical CO_2_ extraction (Sookwong, and Mahatheeranont, 2017). No matter which oil pressing method, it is hard to obtain complete-pesticide-free oil, and trace amount of pesticide residues may be still detectable in extracted edible oils. To remove as more as possible the pesticide residue in oil, different counties have made different criterion for limiting the pesticides. Among them, the European Union is the organization with the strictest detection criteria. Therefore, the EU standards can be used to evaluate pesticide residues after processing.

## Prospective

**Figure 5:**
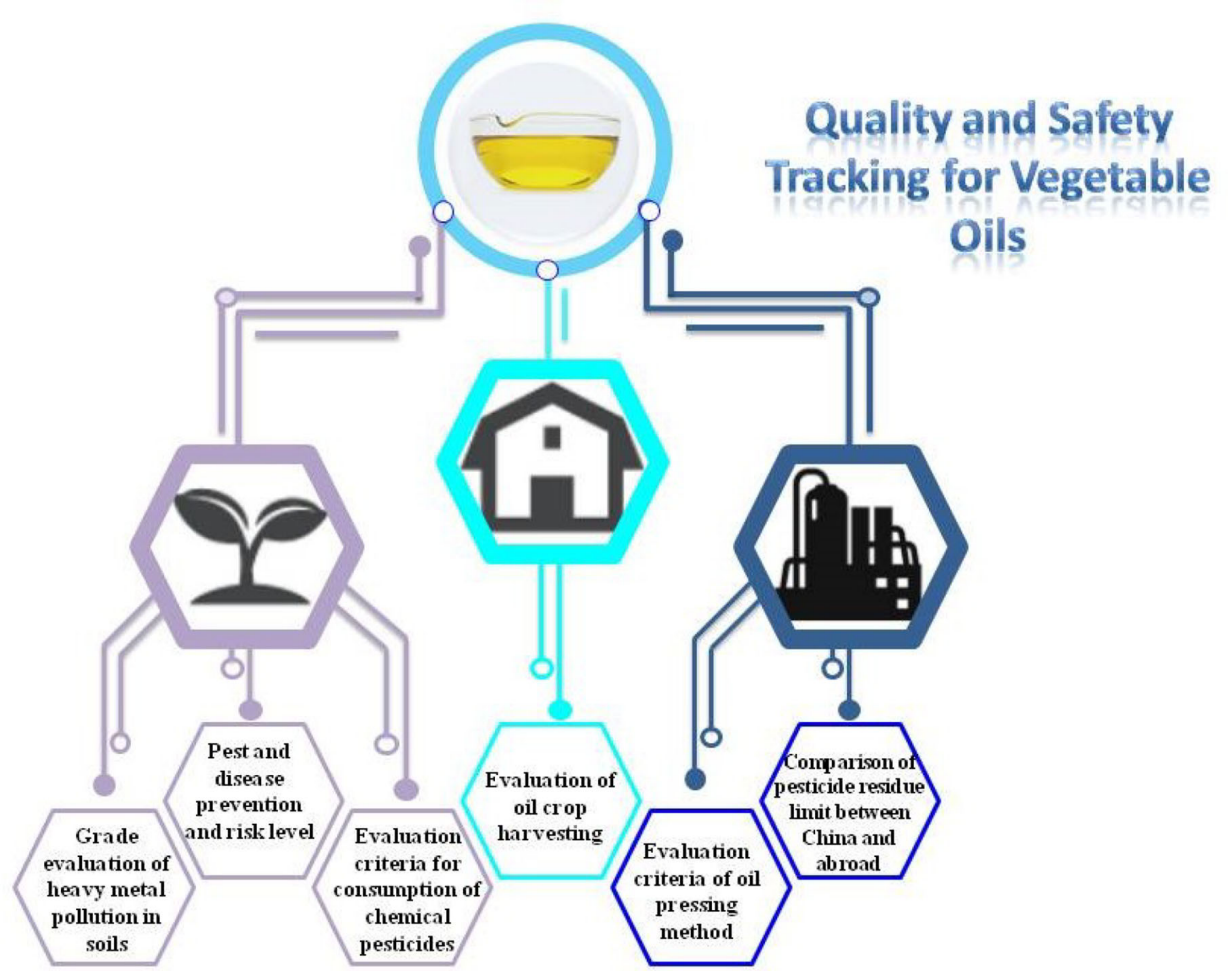
The quality and safety of EPOs monitored during the whole industrial chain.

The quality and safety of edible oil are related to human health, attracting the attention of all human beings. Its evaluation depends on different testing data, testing depends on instruments to complete, and advanced scientific instruments are the material basis to promote more accurate testing. At present, compared with developed countries, developing countries’ technology and means of detecting pesticide residues and harmful substances in EPOs remain slightly inferior. Recently, a new method, the quick, easy, cheap, effective, rugged, and safe (QuEchERS) procedure using high performance liquid chromatography-tandem mass spectrometry (HPLC-MS/MS), has been applied in the detection of pesticide residues (Song et al., 2018). This method is simple, fast, effective, and has good purification effect (Deng et al., 2018). It is also necessary to develop rapid detection techniques for the detection of different hazardous substances in raw materials and EPOs. In the future, with the continuous development of science and technology, more advanced instruments can be used for detection and a multi-stage evaluation system will be established (**Figure 5**) to deal with the the deteriorating environment, which will help to improve the safety and quality of EPOs.

Of course, the establishment of a multi-stage evaluation system for EPOs requires the joint efforts of all countries in the world. People in different countries have different lifestyles and dietary habits, which is also influenced by religious beliefs and cultural differences. Furthermore, the detection standards of pesticide residues in edible oils in different countries and organizations are various, and subsequently, further communication and improvement is required. This will also be conducive to the global trade of EPOs. In each stage of the edible oil industry, strict monitoring of environmental conditions is required to provide a good and safe environment for the cultivation, harvesting and processing of oil crops, and ultimately to ensure the safety of edible oil production under environmentally friendly conditions.

## Author Contributions

All authors contributed to the article and approved the submitted version.

## Conflict of Interest

The authors declare that the research was conducted in the absence of any commercial or financial relationships that could be construed as a potential conflict of interest.
